# IFN-λ Inhibits Drug-Resistant HIV Infection of Macrophages

**DOI:** 10.3389/fimmu.2017.00210

**Published:** 2017-03-06

**Authors:** Xu Wang, He Wang, Man-Qing Liu, Jie-Liang Li, Run-Hong Zhou, Yu Zhou, Yi-Zhong Wang, Wang Zhou, Wen-Zhe Ho

**Affiliations:** ^1^Department of Pathology and Laboratory Medicine, Temple University Lewis Katz School of Medicine, Philadelphia, PA, USA; ^2^Wuhan Center for Disease Prevention and Control, Wuhan, China; ^3^Wuhan University School of Basic Medical Sciences, Wuhan, China

**Keywords:** IFN-λ, drug-resistant HIV, antiretrovirals, Mx2, tetherin

## Abstract

Type III interferons (IFN-λs) have been demonstrated to inhibit a number of viruses, including HIV. Here, we further examined the anti-HIV effect of IFN-λs in macrophages. We found that IFN-λs synergistically enhanced anti-HIV activity of antiretrovirals [azidothymidine (AZT), efavirenz, indinavir, and enfuvirtide] in infected macrophages. Importantly, IFN-λs could suppress HIV infection of macrophages with the drug-resistant strains, including AZT-resistant virus (A012) and reverse transcriptase inhibitor-resistant virus (TC49). Mechanistically, IFN-λs were able to induce the expression of several important anti-HIV cellular factors, including myxovirus resistance 2 (Mx2), a newly identified HIV post-entry inhibitor and tetherin, a restriction factor that blocks HIV release from infected cells. These observations provide additional evidence to support the potential use of IFN-λs as therapeutics agents for the treatment of HIV infection.

## Introduction

Highly active antiretroviral therapy (HAART) has substantially reduced morbidity and mortality in HIV-infected individuals since its introduction in 1996 (1). Although HAART can suppress plasma viral loads to undetectable levels and improve patient life span (2), a substantial fraction of patients fail therapy and/or experience serious side effects from treatment, accompanied by the emergence of drug-resistant viruses (3, 4). More importantly, patients with HIV-1 infection can harbor the virus in latent reservoirs, such as macrophages, one of the key targets of HIV-1 infection. Studies have shown that the intracellular concentrations of antiretrovirals were significantly lower in macrophages than these in T lymphocytes (5, 6). It is known that macrophages play a crucial role in the host defense against HIV-1 infection, as they produce the multiple intracellular HIV restriction factors (7, 8). As HIV-1 latency is the major obstacle in preventing the eradication of the virus, it is necessary to identify agents that can induce intracellular antiviral factors against HIV-1 in macrophages.

Type III interferons (lambda interferons, IFN-λs) or interleukin-28/29 (IL-28/29) display IFN-like activities (9, 10), although they exert their functions through a receptor distinct from type I IFNs (11, 12). IFN-λ subfamily includes three structurally related cytokine members, IFN-λ1 (IL-29), IFN-λ2 (IL-28A), and IFN-λ3 (IL-28B). IFN-λs could be activated by viral infections or activation of toll-like receptors (13, 14). IFN-λs functionally resemble type I IFNs, the activation of which can trigger antiviral activity *in vitro* (11, 15–18) as well as *in vivo* (19, 20). However, unlike type I IFNs that have receptors expressed on many cell types, including the cells in brain, the expression pattern of IFN-λ receptors is more limited to specific cell types (17, 21–24). Thus, IFN-λs have fewer side effects than type I IFNs. The clinical importance of IFN-λs as novel antiviral therapeutic agents has recently become apparent. Several studies (12, 25–27) reported that the endogenous IFN-λ system is associated with treatment-induced clearance of hepatitis C virus (HCV). Furthermore, pegylated IFN-λ works as well as pegylated IFN-α for treating chronic hepatitis C (28–31), but with less side effects in several clinical trial studies. While it has been reported that IFN-λs could inhibit HIV replication in macrophages (17, 18) and CD4^+^ T cells (32), it is unclear whether IFN-λs can inhibit HIV infection with drug-resistant strains. In the present study, we investigated the antiviral effect of IFN-λs on antiretroviral-drug-resistant HIV strains in primary human macrophages. We also determined whether IFN-λs have synergistic effect on anti-HIV activity of antiretroviral drugs in infected macrophages.

## Materials and Methods

### Monocyte and Macrophage Culture

Purified human peripheral blood monocytes were purchased from Human Immunology Core at the University of Pennsylvania (Philadelphia, PA, USA). The Core has the Institutional Review Board approval for blood collection from healthy donors. Monocytes were plated in 48-well culture plates (Corning CellBIND Surface, Corning Incorporated, Corning, NY, USA) at a density of 0.25 × 10^6^ cells/well or 96-well culture plates (Corning CellBIND Surface, Corning Incorporated, Corning, NY, USA) at a density of 10^5^ cells/well in the DMEM containing 10% FCS (33, 34). The medium was half-changed every 2 days. Monocytes differentiated to macrophages after *in vitro* cultured for 5–7 days. We used 7-day-cultured macrophages for experiments of this study.

### HIV Strains and Other Reagents

Based on their differential use of the major HIV receptors (CCR5 and CXCR4), HIV isolates are classified to R5, X4, and R5X4 strains (35). HIV Bal strain (R5 tropic), AZT-resistant HIV A012 G691-6 strain (R5X4 tropic) (36) and the antiretroviral drugs (AZT, efavirenz, indinavir, and enfuvirtide) were obtained from the AIDS Research and Reference Reagent Program at NIH (Bethesda, MD, USA). Reverse transcriptase (RT) inhibitor-resistant HIV TC49 strain (R5 tropic) was kindly provided by Dr. David Katzenstein (Stanford University, Palo Alto, CA, USA). Recombinant human IFN-λ1 and IFN-λ2 were purchased from PeproTech Inc. (Rocky Hill, NJ, USA). Recombinant human IFN-λ3 was purchased from R&D Systems, Inc. (Minneapolis, MN, USA).

### IFN-λs and/or Anti-HIV Drug Treatment and HIV Infection

For infection with the resistant HIV strains, 7-day-cultured macrophages (10^5^ cells/well in 96-well plates) were incubated with or without IFN-λ1, λ2, or λ3 (100 ng/ml each) and/or anti-HIV drugs: azidothymidine (AZT) 10^−11^M; efavirenz 10^−10^M; indinavir 10^−15^M, and enfuvirtide 10^−8^M for 24 h. Cells were then infected with different strains of HIV (6 ng p24/well) for 2 h. After washed three times with plain DMEM, cells were cultured with fresh 10% DMEM containing IFN-λs and/or antiretroviral drugs. For HIV Bal infection, culture supernatant was harvested at day 8 postinfection for RT and p24 assays. Infected and untreated cells served as controls. HIV Gag gene expression in infected cells was also examined at day 8 postinfection. For anti-HIV drug-resistant virus (A012 G691-6 or TC49) infection, culture supernatant was harvested for HIV p24 protein by ELISA at days 3, 5, 7, and 10 postinfection. The cell cultures were replaced with the fresh media supplemented with IFN-λ1, λ2, or λ3 and/or the antiretrovirals every 2–3 days. The culture supernatant collected at day 10 postinfection was also subjected to RT assay.

### HIV RT and p24 ELISA Assays

HIV RT activity was determined based on the technique (37) with modifications (38, 39). For HIV p24 assay, the cultured supernatant was analyzed ELISA as described in the protocol provided by the manufacturer (Chiron Corp., Emeryville, CA, USA).

### RNA Extraction and Real-time RT-PCR

RNA was extracted from cell cultures with Tri-Reagent (Molecular Research Center, Cincinnati, OH, USA) as previously described (40, 41). Total RNA (1 μg) was subjected to RTusing the RT system (Promega, Madison, WI, USA) for 1 h at 42°C. The reaction was terminated by incubating the reaction mixture at 99°C for 5 min, and the mixture was then kept at 4°C. The resulting cDNA was then used as a template for real-time PCR quantification. Real-time PCR was performed with 1/10 of the cDNA with the iQ SYBR Green Supermix (Bio-Rad Laboratories, Hercules, CA, USA) as previously described (41–43). The oligonucleotide primers were synthesized by Integrated DNA Technologies, Inc. (Coralville, IA, USA) and sequences will be available upon request. For the Gag gene expression, the specific oligonucleotide primers are listed as follows: Gag gene primer: 5′-ATAATCCACCTATCCC-AGTAGGAGAAA-3′ (SK38) and 5′TTTGGTCCTTGTCTTATGTCCAGAATGC-3′ (SK39) (44). For the tetherin gene expression, the specific oligonucleotide primers are listed as follows: 5′-AAGAAAGTGGAGGAGCTTTGAGG-3′ (Sense) and 5′-CCTGGTTTTCTCTTCTCAGT-CG-3′ (anti-sense). For the Mx2 gene expression, the specific oligonucleotide primers are listed as follows: 5′-CAGCCACCACCAGGA AACA-3′ (Sense) and 5′-TTCTGCTCGTACTGGCTGTACAG-3′ (anti-sense). The data were normalized to glyceraldehyde-3-phosphate dehydrogenase (GAPDH, primers are 5′-GGTGGTCTCCTCTGACTTC AACA-3′ for sense and 5′-GTTGCTGTAGCCAAATTCGTTGT-3′ for anti-sense, respectively) and presented as the change in induction relative to that of untreated control cells.

### Flow Cytometric Analysis

Cultured macrophages (2.5 × 10^5^ cells/well in 48-well plate) were incubated with or without IFN-λ 1, 2, or 3 (100 ng/ml) for 24 h. Cells were then harvested, washed twice with phosphate-buffered saline containing 1% fetal bovine serum, incubated with PE-conjugated anti-human tetherin (CD317; BioLegend, San Diego, CA, USA) on ice in dark for 30 min. Unstained or isotope-matched mouse immunoglobulin G1-stained cells were included as a negative control. Stained cells were acquired by fluorescence-activated cell sorting (FACSCalibur; BD Biosciences, San Jose, CA, USA) and analyzed using FlowJo software (Tree Star Inc., Ashland, OR, USA).

### Western Blotting for Cell Lysates

The expression of the Mx2 and tetherin were evaluated by immunoblot analysis. Following incubation with polyclonal antibodies to Mx2 (Novus, Littleton, CO, USA) or polyclonal rabbit anti-BST-2 (tetherin) serum (AIDS Research and Reference Program, Bethesda, MD, USA) and extensive washing in PBS containing 0.05% Tween-20, the membranes were incubated with horseradish peroxidase-conjugated IgG (Pierce, Chester, UK) for 1 h at room temperature. The membranes were further washed in PBS. The immunoblots were visualized by enhanced chemiluminescence detection (Amersham, Bucks, UK).

### Statistical Analysis

For comparison of the mean of two groups, statistical significance was assessed by Student’s *t*-test. One-way ANOVA were used for comparison of result between the different groups (multiple comparisons). All graphs were generated and statistical analyses were performed with GraphPad InStat Statistical Software (GraphPad Software Inc., San Diego, CA, USA), and the data are presented as mean ± SD. Statistical significance was defined as *p* < 0.05.

## Results

### IFN-λs Enhance Anti-HIV Activity of Antiretrovirals

**Figure 1:**
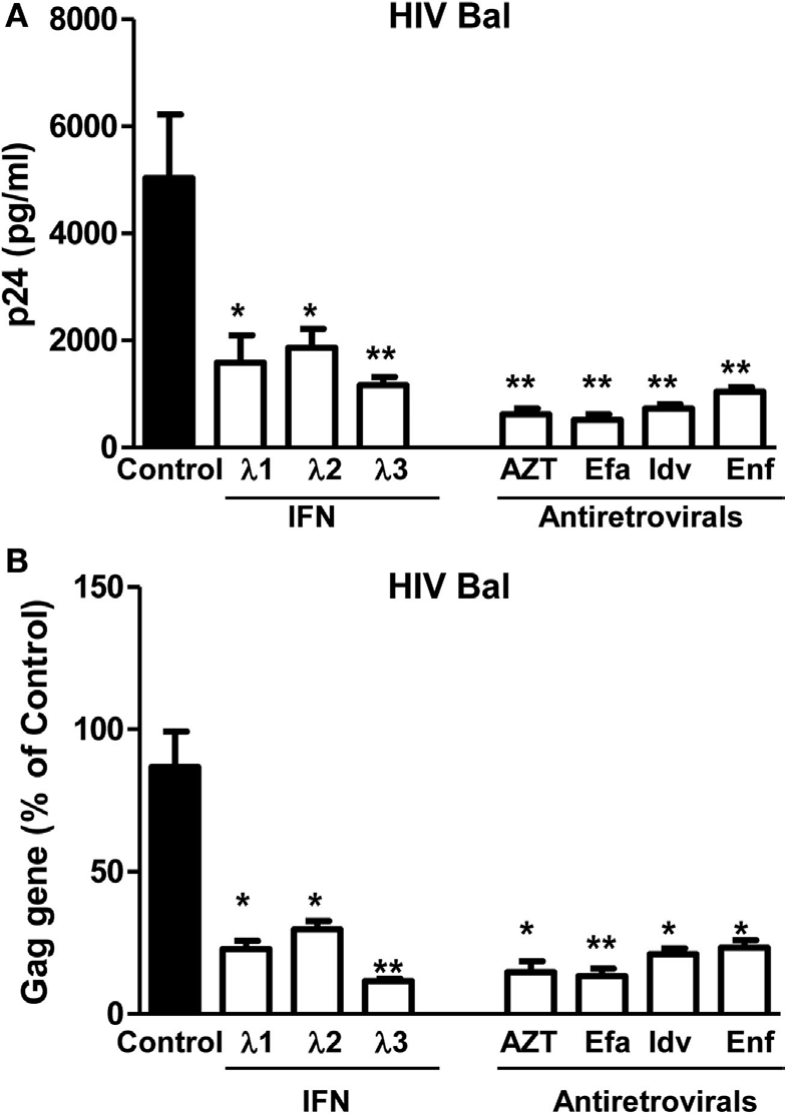
**Effect of IFN-λs on HIV (Bal strain) infection of macrophages**. Seven-day-cultured macrophages were incubated in the presence or absence of IFN-λs (1, 2, or 3; 100 ng/ml each) or classic antiretrovirals at indicated concentrations (AZT: 10^−11^M; efavirenz: 10^−10^M; indinavir: 10^−15^M; and emfuviride: 10^−8^M) for 24 h and then infected with HIV Bal strain. HIV-1 p24 production and Gag gene expression was determined at day 8 postinfection. **(A)** The cell culture supernatant was subjected to ELISA assay to detect HIV p24. **(B)** Total RNA from cells was subjected to HIV Gag gene expression by real-time RT-PCR. The data are expressed as RNA levels relative (percent) to the control (without treatment, which is defined as 100%). The results shown are the mean ± SD from three independent experiments with triplicate wells (*p < 0.05, **p < 0.01, IFN-λ, or antiretroviral vs. control; Efa, efavirenz; Idv, indinavir; Enf, enfuvirtide).

**Figure 2:**
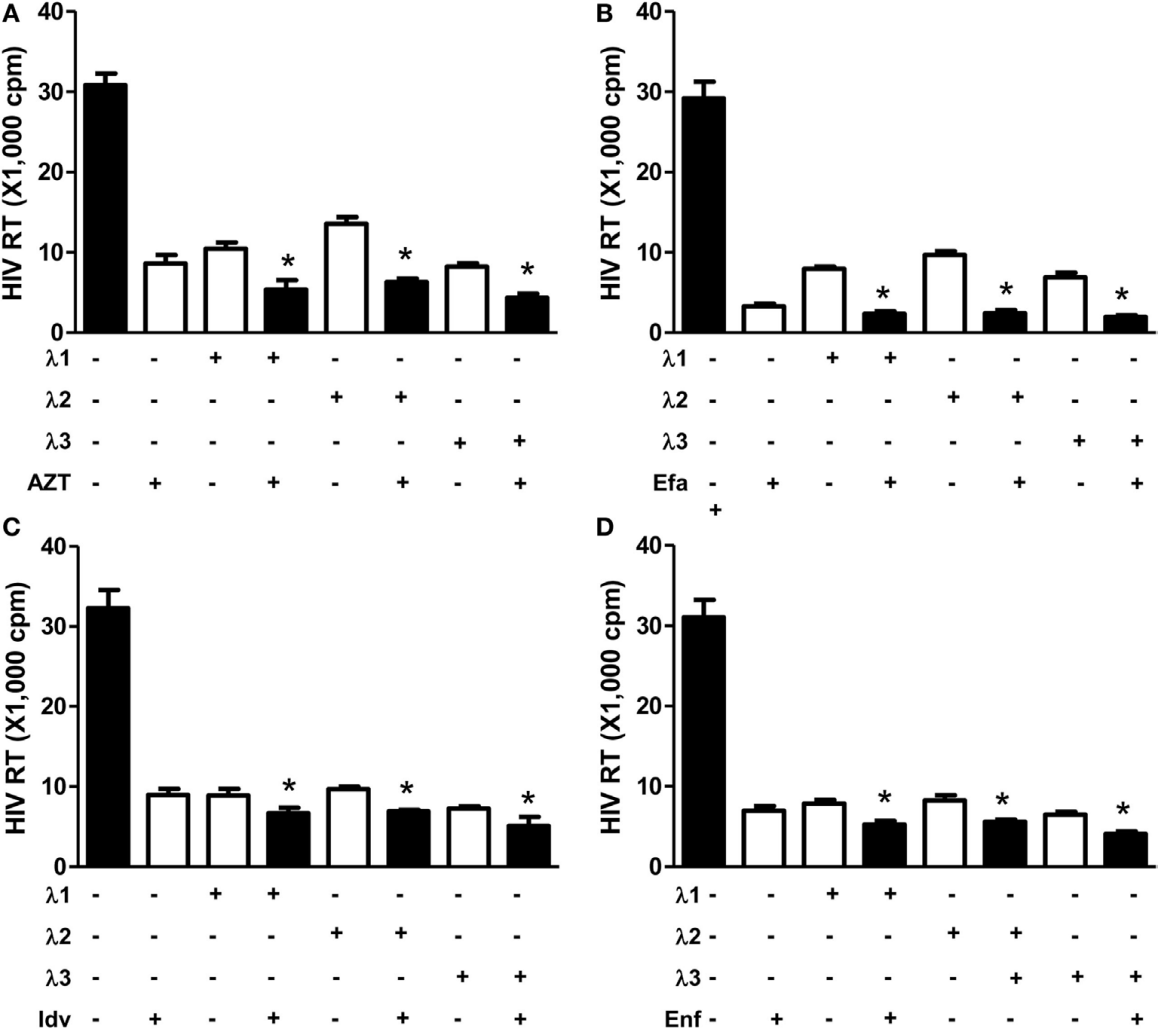
**Effect of IFN-λs and/or antiretrovirals on HIV Bal infection of macrophages**. Seven-day-cultured macrophages were incubated in the presence or absence of IFN-λs (1, 2, or 3; 100 ng/ml each) or classic antiretrovirals **(A)** AZT: 10^−11^M; **(B)** efavirenz: 10^−10^M; **(C)** indinavir: 10^−15^M; and **(D)** emfuviride: 10^−8^M) for 24 h and then infected with HIV Bal strain. HIV RT activity was determined at day 8 postinfection. The data shown are the mean ± SD from three independent experiments with triplicate wells (*p < 0.05, **p < 0.01, IFN-λ+ antiretrovirals vs. antiretrovirals only; Efa, efavirenz; Idv, indinavir; Enf, enfuvirtide).

We first determined the effect of IFN-λs and/or the antiretrovirals (AZT, efavirenz, indinavir, and enfuvirtide) on HIV Bal infection of macrophages. IFN-λs (1, 2, or 3) or the antiretrovirals (AZT, efavirenz, indinavir, and enfuvirtide) significantly inhibited the expression of HIV p24 antigen (Figure 1A) and Gag gene (Figure 1B) in macrophages. IFN-λs (1, 2, or 3) also enhanced the anti-HIV (Bal) effect of AZT (Figure 2A), efavirenz (Figure 2B), indinavir (Figure 2C), and enfuvirtide (Figure 2D).

### IFN-λs Inhibit Drug-Resistant HIV Infection of Macrophages

**Figure 3:**
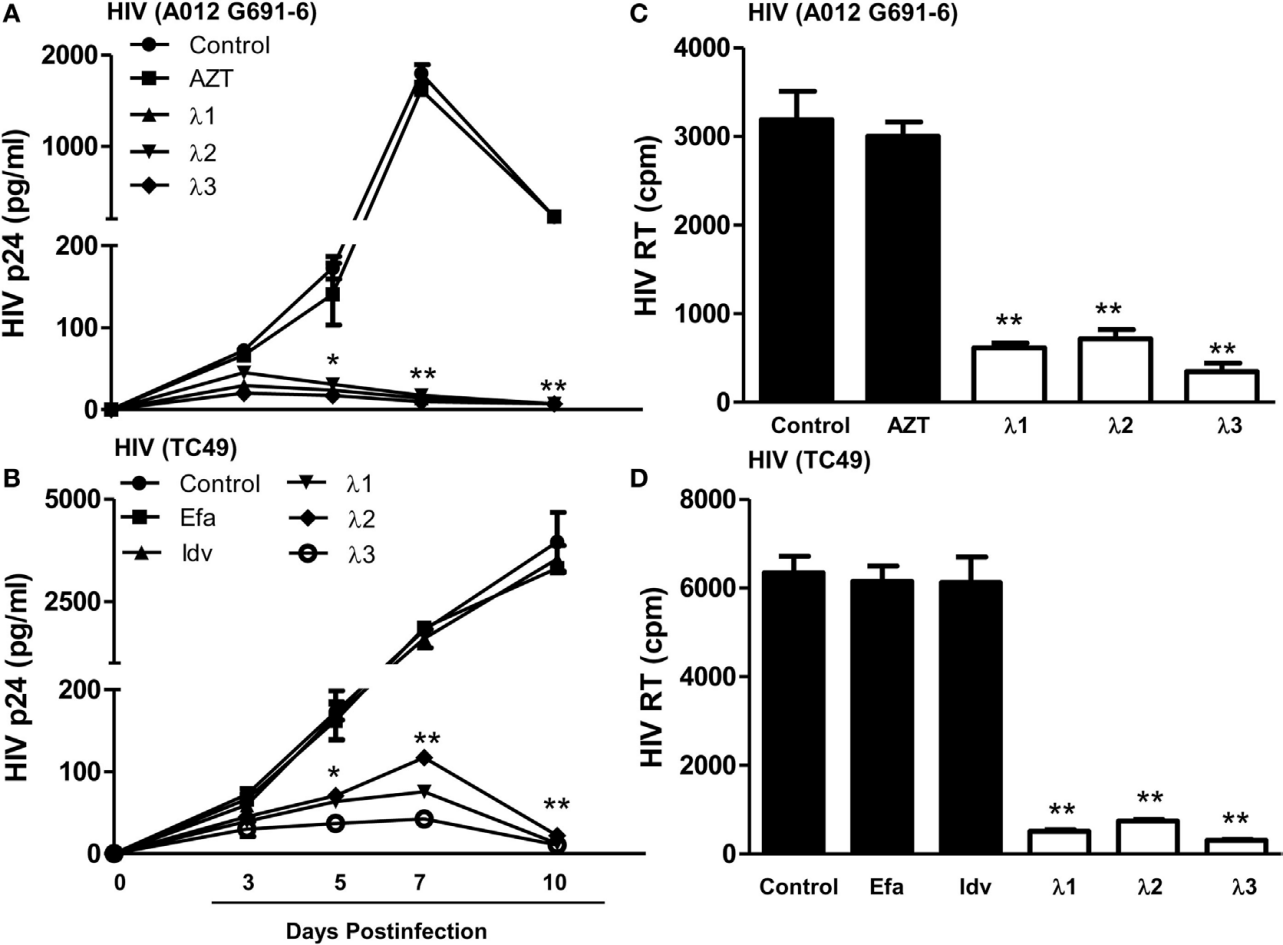
**Effect of IFN-λs on drug-resistant HIV infection of macrophages**. Seven-day-cultured macrophages were incubated in the presence or absence of IFN-λs (1, 2, or 3; 100 ng/ml each) for 24 h and then infected for 6 h with two drug-resistant viruses (A012 G691-6 or TC49). The HIV p24 antigen **(A,C)** was detected at indicated time points post HIV infection using a commercially available ELISA kit, and HIV RT activity **(B,D)** was assayed from the culture supernatant at day 10 postinfection. The data shown are the mean ± SD from three independent experiments with triplicate wells (*p < 0.05, **p < 0.01, IFN-λ vs. control; Efa, Efavirenz, 10^−10^M; Idv, Indinavir, 10^−15^M; AZT 10^−11^M).

We next examined whether IFN-λs (1, 2, or 3) can inhibit drug-resistant HIV infection of macrophages. While AZT had little effect on AZT-resistant HIV strain (A012) infection (Figures 3A,C IFN-λs 1, 2, or 3) potently suppressed infection of macrophages by the AZT-resistant HIV strain (A012) (Figures 3A,C). Similarly, IFN-λ 1, 2, or 3) could suppress RT inhibitor-resistant HIV (TC49) infection of macrophages. In contrast, the RT inhibitors (efavirenz) could not inhibit TC49 strain infection of macrophage (Figures 3B,D).

### IFN-λs Upregulate Tetherin

**Figure 4:**
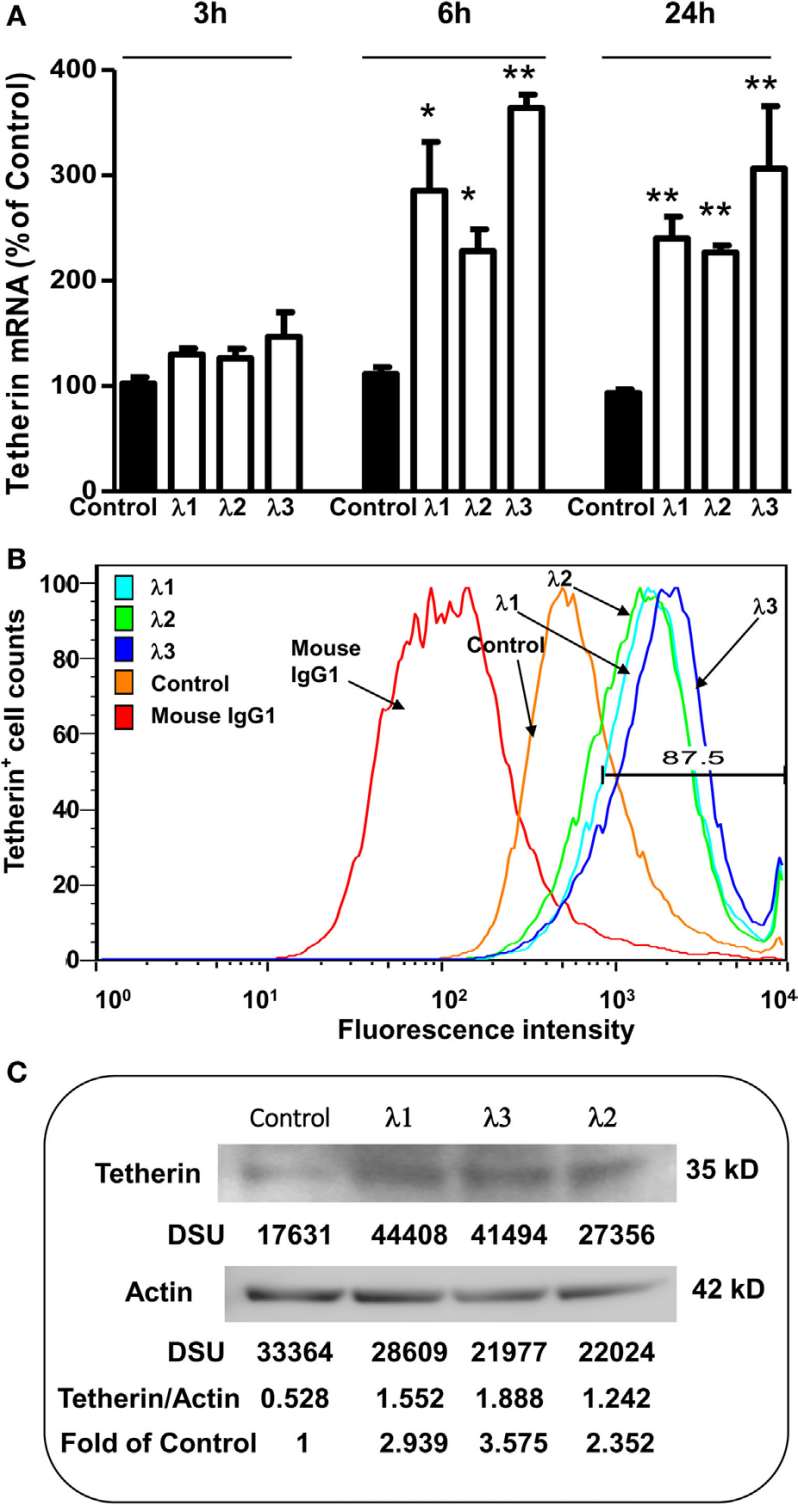
**Effect of IFN-λs on tetherin expression**. **(A)** Tetherin messenger RNA (mRNA) expression. Seven-day-cultured macrophages were incubated in the presence or absence of IFN-λs (1, 2, or 3; 100 ng/ml) for 3, 6, or 24 h. Total RNA was extracted from cells and then the real-time RT-PCR was performed to determine the induced mRNA expression of tetherin and GAPDH. The data are expressed as mRNA levels for tetherin relative (fold) to the control (without IFN-λ treatment, which is defined as 1). The results shown are the mean ± SD from three independent experiments with triplicate wells (**p < 0.01; *p < 0.05; IFN-λ vs. control). **(B)** Tetherin protein expression (flow cytometry). Seven day-cultured macrophages were treated with or without IFN-λs (1, 2, or 3; 100 ng/ml each) for 24 h. Cells were stained with fluorescence-conjugated anti-human tetherin (CD317) antibody and analyzed for tetherin expression by flow cytometry. The isotope control is staining with isotope-matched antibody (immunoglobulin G1). A representative histogram graph was shown. **(C)** Tetherin protein expression (Western blot). Seven day-cultured macrophages were treated with or without IFN-λ (1, 2, or 3; 100 ng/ml each) for 24 h. Total protein exacted from macrophages was subjected to Western blot assay using antibody against tetherin and actin. The inserts below the panels show the signal intensities [density scan unit (DSU)] of protein bands of the representative blot, expressed as densitometry scanning units. The results shown are representative of three independent experiments.

Tetherin, an important IFN-α inducible cellular restriction factor, has been shown to inhibit HIV infection of host cells by preventing release of virus from an infected cell (45, 46). Thus, we examined whether IFN-λ treatment of macrophages can induce the tetherin expression. As shown in Figure 4, IFN-λ treatment of macrophages significantly increased the tetherin expression at both messenger RNA (mRNA) (Figure 4A) and protein (Figures 4B,C) levels.

### IFN-λs Enhance Mx2

**Figure 5:**
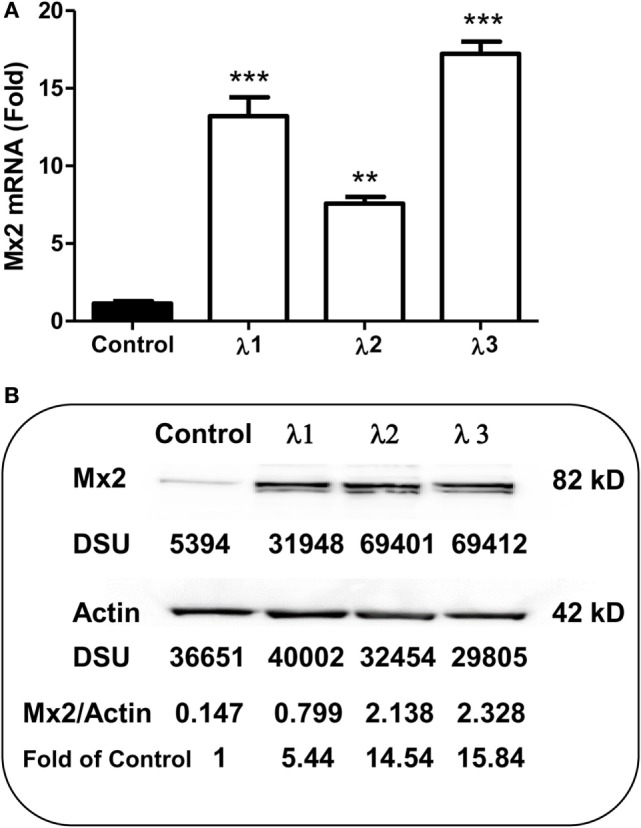
**Effect of IFN-λs on Mx2 expression**. **(A)** Mx2 messenger RNA (mRNA) expression. Seven-day-cultured macrophages were incubated in the presence or absence of IFN-λs (1, 2, or 3; 100 ng/ml each) for 24 h. Total RNA extracted from cells was subjected to the real-time RT-PCR was performed for Mx2 mRNA and GAPDH. The data are expressed as Mx2 mRNA levels relative (fold) to the control (without IFN-λ treatment, which is defined as 1). The results shown are the mean ± SD from three independent experiments with triplicate wells (**p < 0.01; *p < 0.05; IFN-λ vs. control). **(B)** Mx2 protein expression (Western blot). Seven day-cultured macrophages were treated with or without IFN-λ (1, 2, or 3; 100 ng/ml each) for 24 h. Total protein exacted from macrophages was subjected to Western blot assay using antibody against Mx2 and actin. The inserts below the panels show the signal intensities [density scan unit (DSU)] of protein bands of the representative blot, expressed as densitometry scanning units. The results shown are representative of three independent experiments.

As an IFN-α-inducible cellular factor, Mx2 has recently been identified to inhibit HIV at post-entry level (47–49). Mx2 could abolish capsid-dependent nuclear import of subviral complexes (41–43). We thus examined whether IFN-λs can induce Mx2 expression in macrophages. As shown in Figure 5, IFN-λ treatment of macrophages significantly upregulated the Mx2 expression at both mRNA (Figure 5A) and protein (Figure 5B) levels.

## Discussion

To find new antiretroviral agents remains to be an important area of anti-HIV studies. Our earlier studies showed that IFN-λs could inhibit *in vitro* HIV infection/replication (17, 18, 50). IFN-λs are a class of recently identified members of IFN family, including three IFN-λ (lambda) molecules called IFN-λ1, IFN-λ2, and IFN-λ3 (also called IL-29, IL-28A, and IL-28B, respectively) (51). IFN-λs bind to their own distinctive receptor complex, IL-10Rβ and IL-28Rα, which activates janus kinase/signal transducers and activators of the transcription (JAK/STAT) signaling pathway, resulting in the phosphorylation of STAT proteins and forming of IFN-stimulated gene factor 3 complex (11, 15, 52).

While the IL-10Rβ shows a broad expression pattern (53), expression of the IFN-λ receptor subunit IL-28Rα is much more restricted (11, 54, 55). Earlier analysis of the expression pattern of IL-28Rα in human tissues showed that IL-28Rα mRNA levels were highest in the lung, heart, liver, and prostate, while low mRNA levels were detected in the central nervous system, bone marrow, testis, uterus, and skeletal muscle (22, 54, 55). A few immune cells express IL-28Rα especially at the mRNA level (e.g., B cells, macrophages, and plasmacytoid DCs), but conflicting protein expression data are reported in the literature (17, 22, 54–57). Although some evidence indicates that IFN-λ receptor expression on peripheral leukocytes is not functional (22), other evidence shows clear antiviral innate defense in some of these cells, and IFN-λ signals stimulate monocytes and macrophages to produce IL-6, IL-8, and IL-10 (58).

Studies from different investigators have demonstrated that IFN-λs have the ability to inhibit the replication of a number of viruses, including HCV and hepatitis B virus (16, 59), cytomegalovirus (60), Apeu virus (61), herpes simplex virus type 2 (HSV-2) (19), encephalomyocarditis virus (11), vesicular stomatitis virus (60), West Nile virus (62), and dengue virus (63). IFN-λs also had antiviral effect *in vivo* (19, 64, 65). Recent *in vivo* studies with mice showed that IFN-λs had the ability to reduce hepatic viral titer of HSV-2 and completely blocked HSV-2 replication in vaginal mucosa (19). IFN-λs contribute to innate immunity of mice against influenza A virus (66, 67). In addition, their role in direct antiviral effects *in vivo* has also been demonstrated in IL-28RA and STAT1 knockout animals, where a significant increase in influenza A virus replication was observed (66–68). Others and we have shown that IFN-λs could inhibit HIV infection of CD4^+^ T cells (32) and macrophages (17, 18, 50). Mechanistically, IFN-λ1 and IFN-λ2 were able to induce the intracellular expression of type I IFN, CC chemokines (the ligands for CCR5), and APOBEC3G/3F, the cellular HIV restriction factors (17). In addition, we demonstrated that IFN-λ3 could induce multiple antiviral cellular factors (ISG56, MxA, OAS-1) (18). We also showed that all three IFN-λs could induce the expression of pattern recognition receptors in macrophages (50). The *in vivo* production of IFN-λ1 also was monitored in HIV-infected patients. Tian et al. found that the plasma IFN-λ1 levels were increased along with the depletion of CD4^+^ T cells in HIV-1-infected patients, but the elevated IFN-λ1 showed limited repression of viral production (32).

In the present study, we further examined the anti-HIV activity of IFN-λs. We showed that all three IFN-λs not only inhibited drug-resistant virus replication (Figure 3) but also enhanced the anti-HIV effect of commonly used antiretrovirals (Figures 1 and 2), including zidovudine (AZT, a nucleoside RT inhibitor), efavirenz (a non-nucleoside RT inhibitor), indinavir (protease inhibitor), and enfuvirtide (HIV fusion inhibitor). In addition to the reported mechanisms involved in IFN-λ-mediated HIV inhibition (17, 18, 50): the induction of extracellular factors, e.g., CC chemokines that block HIV entry into macrophages, and the activation of intracellular innate immunity, e.g., the induction of type I IFNs and APOBEC3G/F, we demonstrated that IFN-λ treatment of macrophages induced the expression of tetherin, a cellular factor that can block HIV infection by preventing virus release from infected cells (Figure 4). In addition, IFN-λs also enhanced the expression of Mx2 (Figure 5), a newly identified HIV post-entry inhibitor that can abolish capsid-dependent nuclear import of subviral complexes (41–43). These anti-HIV cellular factors are the contributors for IFN-λ-mediated anti-HIV activity. These findings in conjunction with our previous observations (17, 18, 50) indicate that IFN-λs are attractive alternative for HIV treatment, as it would be extremely difficult for HIV to develop resistance to IFN-λs that can suppress the virus at various steps of its replication. However, further studies are necessary to determine the impact of IFN-λs on drug-resistant HIV strains in *ex vivo* and *in vivo* systems. These additional studies shall explore the clinical potential for developing IFN-λs-based therapy for HIV/AIDS.

## Ethics Statement

In this *in vitro* study, we obtained primary human monocytes from the Immunology Core at the University of Pennsylvania School of Medicine. The Core has the Institutional Review Board approval for blood collection from healthy donors. Anyone who obtains human cells from the Core is considered as secondary use of de-identified human specimens, which does not subject to human subject review by both NIH and IRB.

## Author Contributions

XW and W-ZH designed the study. XW, M-QL, YZ, R-HZ, and Y-ZW performed the experiments. J-LL supplied reagents needed for this study. XW, M-QL, and YZ analyzed and interpreted the data and wrote the manuscript. HW, WZ, and W-ZH reviewed and revised the manuscript. All the authors have read, reviewed, and edited the manuscript and agreed for submission to this journal.

## Conflict of Interest Statement

The authors declare that the research was conducted in the absence of any commercial or financial relationships that could be construed as a potential conflict of interest.
